# Can micro-expressions be used as a biomarker for autism spectrum disorder?

**DOI:** 10.3389/fninf.2024.1435091

**Published:** 2024-10-03

**Authors:** Mindi Ruan, Na Zhang, Xiangxu Yu, Wenqi Li, Chuanbo Hu, Paula J. Webster, Lynn K. Paul, Shuo Wang, Xin Li

**Affiliations:** ^1^Lane Department of Computer Science and Electrical Engineering, West Virginia University, Morgantown, WV, United States; ^2^Department of Radiology, Washington University, St. Louis, MO, United States; ^3^Department of Computer Science, University at Albany, Albany, NY, United States; ^4^Department of Chemical and Biomedical Engineering, West Virginia University, Morgantown, WV, United States; ^5^Division of the Humanities and Social Sciences, California Institute of Technology, Pasadena, CA, United States

**Keywords:** autism spectrum disorder (ASD), face videos, micro-expressions, interpretable machine learning, Autism Diagnostic Observation Schedule (ADOS)

## Abstract

**Introduction:**

Early and accurate diagnosis of autism spectrum disorder (ASD) is crucial for effective intervention, yet it remains a significant challenge due to its complexity and variability. Micro-expressions are rapid, involuntary facial movements indicative of underlying emotional states. It is unknown whether micro-expression can serve as a valid bio-marker for ASD diagnosis.

**Methods:**

This study introduces a novel machine-learning (ML) framework that advances ASD diagnostics by focusing on facial micro-expressions. We applied cutting-edge algorithms to detect and analyze these micro-expressions from video data, aiming to identify distinctive patterns that could differentiate individuals with ASD from typically developing peers. Our computational approach included three key components: (1) micro-expression spotting using Shallow Optical Flow Three-stream CNN (SOFTNet), (2) feature extraction via Micron-BERT, and (3) classification with majority voting of three competing models (MLP, SVM, and ResNet).

**Results:**

Despite the sophisticated methodology, the ML framework's ability to reliably identify ASD-specific patterns was limited by the quality of video data. This limitation raised concerns about the efficacy of using micro-expressions for ASD diagnostics and pointed to the necessity for enhanced video data quality.

**Discussion:**

Our research has provided a cautious evaluation of micro-expression diagnostic value, underscoring the need for advancements in behavioral imaging and multimodal AI technology to leverage the full capabilities of ML in an ASD-specific clinical context.

## 1 Introduction

Autism Spectrum Disorder (ASD) is a complex developmental condition characterized by challenges in social interaction, communication, and repetitive behaviors. With its prevalence increasing in recent years, ASD has become a significant public health concern globally. The heterogeneity in symptoms and severity across individuals with ASD makes early and accurate diagnosis crucial for effective intervention and management. Current diagnostic practices for ASD primarily involve standardized assessments such as the Autism Diagnostic Observation Schedule (ADOS) (Lord et al., 1989) and clinical evaluations. These methods are instrumental in identifying the presence of ASD characteristics. However, they also face limitations. The efficacy of these diagnostic tools heavily depends on the expertise of clinicians. It also relies on more observable and overt expressions and behaviors, which can vary significantly among individuals and may be influenced by subjective interpretations. Moreover, the existing diagnostic criteria and methods may not adequately capture the full spectrum of ASD diversity, particularly in cases with subtle or atypical manifestations.

Recent advancements in machine learning have opened new avenues for modeling and diagnosing ASD. Machine learning has been effectively used to analyze eye movement patterns, revealing subtle differences in how individuals with ASD engage with their environment compared to typically developing (TD) peers (Xie et al., 2019; Wang et al., 2015; Ruan et al., 2021; Yu X. et al., 2024). Additionally, machine learning has been applied to analyze body movement patterns, revealing distinct differences in how individuals with ASD exhibit restricted and repetitive behaviors (RRB) in interactive and non-interactive settings compared to TD peers (Ruan et al., 2023; Zunino et al., 2018; Tian et al., 2019). Additionally, machine learning techniques have been employed to refine and enhance the scoring methodologies used in structured diagnostic tools such as the Autism Diagnostic Interview-Revised (ADI-R) and the Autism Diagnostic Observation Schedule (ADOS), leading to more accurate and reliable diagnostic outcomes (Duda et al., 2014; Wall et al., 2012). Furthermore, analyzing home video content through machine learning algorithms has opened new avenues for researchers and clinicians to observe and assess behaviors in naturalistic settings, providing richer, more contextual insights into the spectrum of ASD behaviors (Tariq et al., 2018). Recent work has also explored the use of machine learning to diagnose autism-associated language and speech pattern disorders, identifying distinct linguistic features, such as atypical syntax and semantics, as well as unique speech prosody, which can significantly enhance the sensitivity and specificity of ASD diagnostic tools (Hu et al., 2024a,b). Therefore, machine learning offers a vital and effective method for identifying and comprehending the factors that contribute to atypical behaviors in ASD.

Building on this foundation, our study seeks to expand the application of machine learning further into the realm of facial expression analysis. Facial expressions represent a critical domain of non-verbal communication and emotional expression often disrupted in individuals with ASD. Individuals with ASD often exhibit atypical facial expressions (Webster et al., 2021; Dollion et al., 2022; Loth et al., 2018; Yu H. et al., 2024). It is known that they face challenges in recognition/interpretation/reading and production/expression compared to neurotypicals. Previous studies have demonstrated that individuals with ASD are significantly less accurate in recognizing standard facial expressions of fear, sadness, and disgust. Moreover, they specifically struggle to identify fear from eye expressions and disgust from the mouth, often confusing fearful expressions with anger (Wallace et al., 2008). In a more recent study, misinterpretation of facial expressions by ASD was studied in more detail (Eack et al., 2015). It was reported that adults with ASD notably misinterpreted happy faces as neutral and were significantly more likely than the control group to attribute negative valence to nonemotional faces. These findings suggest a potential negative bias toward the interpretation of facial expressions, which may have implications for behavioral interventions designed to remediate emotion perception in ASD.

Current research on ASD diagnostics has primarily focused on production of macro-level facial expressions. These studies (Zhang et al., 2022; Zhang, 2023; Beary et al., 2020; Akter et al., 2021; Lu and Perkowski, 2021; Jia et al., 2021; Wang et al., 2022, 2024; Wang, 2023) integrating machine learning have predominantly utilized overt facial expressions, often overlooking the rich data present in micro-expressions. In contrast to macro-expressions, which are easily observable and longer-lasting facial expressions typically evaluated in ADOS, *micro-expressions* (Takalkar et al., 2018) are brief, involuntary facial movements that reflect deep, often concealed emotions. Micro-expressions could provide crucial insights into the nuanced emotional landscape of individuals with ASD, which are usually not apparent in their more observable behaviors. These subtle cues might reveal the internal emotional states that are not aligned with the external expressions typically recorded in ASD evaluations. Despite their potential to reveal genuine emotional states, research on micro-expression utility in ASD diagnostics is limited. These rapid facial changes, lasting only a fraction of a second, present a significant challenge in traditional observational settings like the hour-long ADOS sessions. Due to their fleeting nature, the lack of attention to these quick expressions means that current diagnostic practices may overlook an essential aspect of ASD symptomatology, potentially leading to gaps in our understanding and diagnosis of the disorder.

Inspired by recent advances in computer vision (Nguyen et al., 2023) and autism research (Rani and Verma, 2024), this study introduces an innovative approach to ASD diagnostics by leveraging *micro-expressions* as novel features for ML models. By adopting advanced algorithms capable of detecting and analyzing transient and subtle facial cues such as micro-expressions, we aim to uncover patterns that differentiate individuals with ASD from their control counterparts. The hypothesis under testing is: *can facial microexpression be a reliable biomarker for ASD diagnosis?* To the best of our knowledge, this is the first study focusing on micro-level behavioral observations under the context of ASD. Even if patients with ASD possess abnormal micro-expression in theory, designing a computational platform to extract this micro-level feature and test its discriminative power is nontrivial. As the first step, we have leveraged the latest advances in computer vision (e.g., transformer architecture for micro-expression detection) and classical machine learning tools (e.g., support vector machine and permutation tests) to our experimental design. Other system design options, such as closed-loop (end-to-end) optimization of three modules, will be pursued in our next study.

## 2 Materials and methods

### 2.1 Autism Diagnostic Observation Schedule (ADOS-2)

We analyzed videos recorded during the administration of ADOS-2 Module 4 interview. These videos feature structured yet natural dialogues between an interviewer and the participant, capturing various behaviors indicative of ASD in adults. Written informed consent was obtained from all participants, following the ethical guidelines approved by the Institutional Review Boards (IRB) at West Virginia University (WVU), Washington University in St. Louis (WashU), University at Albany (UAlbany), and the California Institute of Technology (Caltech).

ADOS is a pivotal clinical tool and is widely regarded as the gold standard for ASD diagnosis. It employs a series of standardized tasks designed to prompt behaviors critical to autism diagnosis, blending structured activities and informal interactions to observe social and communicative behaviors within a controlled context. During these sessions, ASD experts record participants' reactions to prompts that elicit specific social and communicative responses. These interactions, lasting about an hour, are carefully scored based on a diagnostic algorithm to aid in forming a conclusive diagnosis of ASD.

The ADOS-2 Module 4 consists of 15 interview sections (scenarios), each containing a set of standardized prompts or questions that elicit a range of responses, including verbal communication to physical gestures. In this study, we focused on seven ADOS-2 sections (5–7, 11–14) in which the examiner directly interviews the participant, as these sections are most relevant for our micro-expression analysis. Note that it is far more challenging to extract micro-expression features (e.g., due to extreme pose or partial occlusion) for testing the relevant hypothesis:

The seven interview sections address the following topics:

(1) Current Work or School (Scenario 5): queries about aspects of the participant's life related to work or education;
(2) Social Difficulties and Annoyance (Scenario 6): discussions about social interactions and perceptions thereof;
(3) Emotions (Scenario 7): conversations about situations or objects that evoke various emotions, asking participants to articulate their feelings;
(4) Daily Living (Scenario 11): questions aimed at understanding the participant's living situation and their autonomy;
(5) Friends, Relationships, and Marriage (Scenario 12): assessing the participant's comprehension of these personal connections;
(6) Loneliness (Scenario 13): evaluating the participant's grasp of loneliness;
(7) Plans and Hopes (Scenario 14): exploring the participant's future aspirations and expectations.

#### 2.1.1 Participants

The effectiveness of our model was assessed using two non-public ADOS video datasets from Caltech and WVU, with demographic and clinical characteristics detailed in Table 1.

**Table 1:** Demographic and clinical characteristics of the Caltech and WVU ADOS video dataset.

**Characteristic**	**ASD (mean ±SD)**	**TD (mean ±SD)**	***p*-value**
No. of subjects	33	9	
Sex (M/F)	26/7	3/6	
Hand (left/right)	2/31	1/8	
Age	24.32 ± 5.77	23.6 ± 3.93	0.7932
FSIQ	96.23 ± 13.12	125.4 ± 5.85	0.0004
VIQ	95.38 ± 14.27	121.4 ± 10.38	0.0029
PIQ	97.69 ± 13.68	124.0 ± 8.34	0.0016
ADOS SA	8.29 ± 4.55	–	
ADOS RRB	2.43 ± 1.50	–	
ADOS CSS-SA	6.0 ± 2.52	–	
ADOS CSS-RRB	5.95 ± 2.40	–	
ADOS CSS-AL	5.64 ± 2.79	–	

**Caltech ADOS video dataset**. This dataset involved 33 participants, aged 16–37 years, with a composition of 26 males and seven females, predominantly right-handed (*n* = 31). A subset (*n* = 9) underwent two ADOS interviews ~6 months apart, yielding 42 videos. All were diagnosed with ASD based on the Autism Diagnostic Observation Schedule, Second Edition (ADOS-2) (Lord et al., 1989), confirmed through expert clinical evaluation. Their condition was quantified using calibrated severity scores from ADOS-2: (1) Social Affect (SA): 8.29 ± 4.55; (2) Restricted and Repetitive Behavior (RRB): 2.43 ± 1.50; (3) Severity Score for Social Affect (CSS-SA): 6.0 ± 2.52; (4) Severity Score for Restricted and Repetitive Behavior (CSS-RRB): 5.95 ± 2.40; and (5) Severity Score for SA plus RRB (CSS-All): 5.64 ± 2.79. Furthermore, the ASD group demonstrated cognitive abilities with an average Full-Scale IQ (FSIQ) of 96.23 ± 13.12, Verbal IQ (VIQ) of 95.38 ± 14.27, and Performance IQ (PIQ) of 97.69 ± 13.68, measured using the Wechsler Abbreviated Scale of Intelligence-2 (WASI-II), with a mean age of 24.32 ± 5.77 years. The interview videos were recorded in a quiet room at Caltech with a high-quality video camera, capturing detailed behavioral data, including body movements, facial expressions, and social interactions.

**WVU ADOS video dataset**. This dataset involved nine TD participants, reflecting an average age of 23.6 ± 3.93 years, with a composition of three males and six females, predominantly right-handed (*n* = 8). Participants in the TD group demonstrated cognitive abilities within the normative range, with a WASI-II FSIQ of 125.4 ± 5.85, VIQ of 121.4 ± 10.38, and PIQ of 124.0 ± 8.34. Similar to the Caltech ADOS dataset, the interview videos for the TD group were recorded in a controlled environment at WVU. A high-quality video camera was used in a quiet room setting, ensuring the capture of fine-grained behavioral data. This included detailed observations of body movements, facial expressions, and social interactions.

#### 2.1.2 ADOS coding and items

The ADOS-2 utilizes a systematic coding scheme to assess behaviors indicative of ASD across five key domains, consisting of 32 items: (1) Language and Communication, (2) Reciprocal Social Interaction, (3) Imagination/Creativity, (4) Stereotyped Behaviors and Restricted Interests, and (5) Other Abnormal Behaviors. Our study specifically focused on behaviors coded in the Reciprocal Social Interaction (RSI) domain from the original diagnostic algorithm, exploring how these interactions in the context of micro-expressions could offer valuable insights into subtle social and emotional cues that are often missed in traditional observations. RSI evaluates the individual's capacity for social exchanges characterized by mutual give-and-take and responsiveness—essential aspects of typical social interactions. Assessing this domain is critical for discerning between the ASD and TD participants, and describing the relative severity of social impairments associated with ASD. It is examined through structured and semi-structured tasks designed to evoke social interactions within a controlled environment. The key components related to facial expression in this domain include:

- Eye contact: Evaluates the appropriateness and frequency of eye contact relative to the social context and interaction flow. Poor or atypical use of eye contact is a common feature in ASD.
- Facial expressions used to regulate social interaction: Observes the use and appropriateness of facial expressions during social exchanges. This item assesses whether facial expressions are integrated into communications in a way that seems genuine and appropriate for the context.
- Shared enjoyment in interaction: Looks at the individual's ability to share enjoyment, interests, or achievements with others through typical behaviors such as showing, bringing, or pointing out objects of interest.
- Response to social cues: Measures how the individual responds to social initiations made by others. This includes responses to both verbal and non-verbal cues that require social adaptation and appropriate reactions.
- Quality of social overtures: Assesses the efforts made by the individual to initiate social interactions with others. This includes how the individual approaches and initiates interactions and whether these overtures are appropriate for the social context.

Detailed descriptions of the RSI items are provided in Table 2. Each behavior observed during the ADOS-2 assessment is carefully scored within these domains based on intensity and deviation from typical development. Typically, the item scoring system ranges from 0 to 3, where:

- 0: indicates typical behavior appropriate to the social context.
- 1: mild atypical behavior not entirely consistent with developmental norms but not solely indicative of ASD.
- 2: indicates behavior that is atypical and is often seen in individuals with ASD.
- 3: is used in certain items where the behavior is markedly abnormal and severely affects social interaction or communication.

**Table 2:** Items of Reciprocal Social Interaction (RSI) in ADOS-2 coding scheme.

	**Items**	**Description**
B01	Unusual eye contact	The quality and appropriateness of gaze or eye contact during social interactions.
B02	Facial expressions directed to examiner	Whether the participant's facial expressions are used to communicate affective or cognitive states to the examiner.
B03	Language production and linked nonverbal communication	The extent to which vocalizations are accompanied by subtle shifts in gaze, facial expression, and gestures.
B04	Shared enjoyment in interaction	The participant's directed pleasure during any tasks or conversation.
B05	Communication of own affect	The participant's ability to convey a range of his or her own emotions using words and facial expressions, tone of voice, vocalization, and/or gestures.
B06	Comments on others' emotions/empathy	The participant's ability to communicate their recognition, understanding, and/or response to the emotions of others or characters, whether real or depicted in stories or tasks.
B07	Insight into Typical Social Situations and relationships	The participant's ability to spontaneously provide examples demonstrating insight into the nature of social relationships, encompassing both ongoing relationships like friendships or marriage and situational interactions such as getting along with peers or co-workers.
B08	Responsibility	The participant's references to and descriptions of being responsible for his or her own actions in typical daily living situations.
B09	Quality of social overtures	The quality of the participant's efforts to initiate social interaction with the examiner.
B10	Amount of social overtures/Maintenance of Attention	The number of participants' attempts to get, maintain, or direct the examiner's attention.
B11	Quality of social response	The participant's social responses throughout the ADOS-s evaluation.
B12	Amount of reciprocal social communication	The frequency with which reciprocal interchanges occur during the course of the ADOS-2 evaluation, using any mode of communication.
B13	Overall quality of rapport	Rate that reflects the examiner's overall judgment of the rapport established with the participant during the ADOS-2 evaluation.

### 2.2 Using micro-expressions for classification

**Figure 1:**
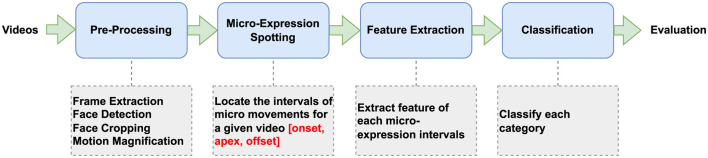
Overview of our video-based ASD classification based on micro-expression extraction.

Our approach to diagnosing ASD employed a structured framework encompassing three critical stages: spotting micro-expressions, extracting features from micro-expressions, and classifying micro-expressions. Figure 1 shows the framework of our micro-expression classification model. Initially, videos underwent a preprocessing step where frames were extracted, faces were detected and cropped, and the motion was magnified to accentuate relevant facial movements. This stage ensured that the data was optimized for accurately detecting micro-expressions. Subsequently, we applied micro-expression spotting techniques to the preprocessed video, locating the segments that potentially contained micro-movements indicative of underlying emotional states. This critical step involved identifying the onset and apex of these micro-expressions for each video segment. Once the micro-expression intervals were identified, the next step in our framework was to extract features from these segments. The extraction process was designed to distill each micro-expression into a set of features, preparing the data for classification. These features served as the basis for the next step, where a machine learning classifier was employed to categorize each segment. This classification determined whether the segment's features corresponded to ASD-specific behaviors or to those of TD individuals.

#### 2.2.1 Data preprocessing

To ensure consistency and quality across all facial data used in our study, we standardized the resolution of facial regions in each video frame. Each frame was meticulously cropped and resized to a uniform dimension of 128 × 128 pixels. The cropping process began by identifying a square bounding box around the face in each video's initial (reference) frame. We selected an intentionally larger bounding box to ensure complete facial inclusion throughout the video, accommodating any participant movements.

**Figure 2:**
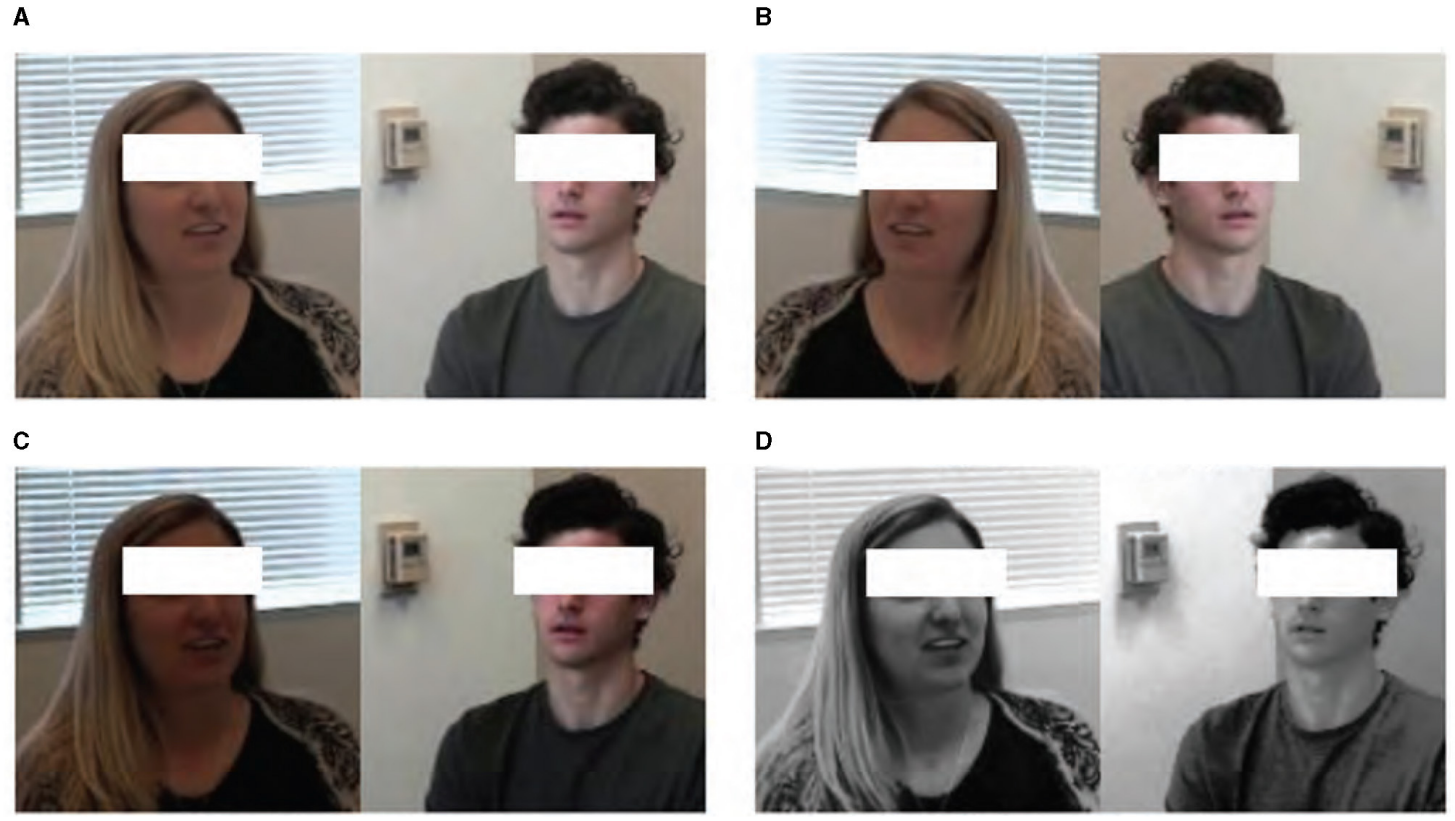
Data augmentation results of **(A)** raw data by **(B)** horizontal flipping, **(C)** brightness changing, and **(D)** histogram equalization.

To address the challenge of data imbalance between ASD and TD—an issue that can significantly affect the performance of machine learning models—we employed data augmentation techniques on the cropped facial frames. This approach effectively increased the size and variability of our control group dataset, thereby enhancing the model's ability to generalize across different individuals and conditions. Augmentation techniques included horizontal flipping, brightness adjustments, and histogram equalization, each carefully chosen to simulate a wider range of potential recording conditions without distorting the inherent facial expressions. Initially, the ASD group contained 42 videos, while the TD group dataset comprised only nine videos; however, our augmentation efforts expanded this to 36 videos. This expansion is crucial for ensuring a robust comparative analysis between the groups. These augmentation strategies are visually summarized in Figure 2.

#### 2.2.2 Spotting micro-expressions

**Figure 3:**
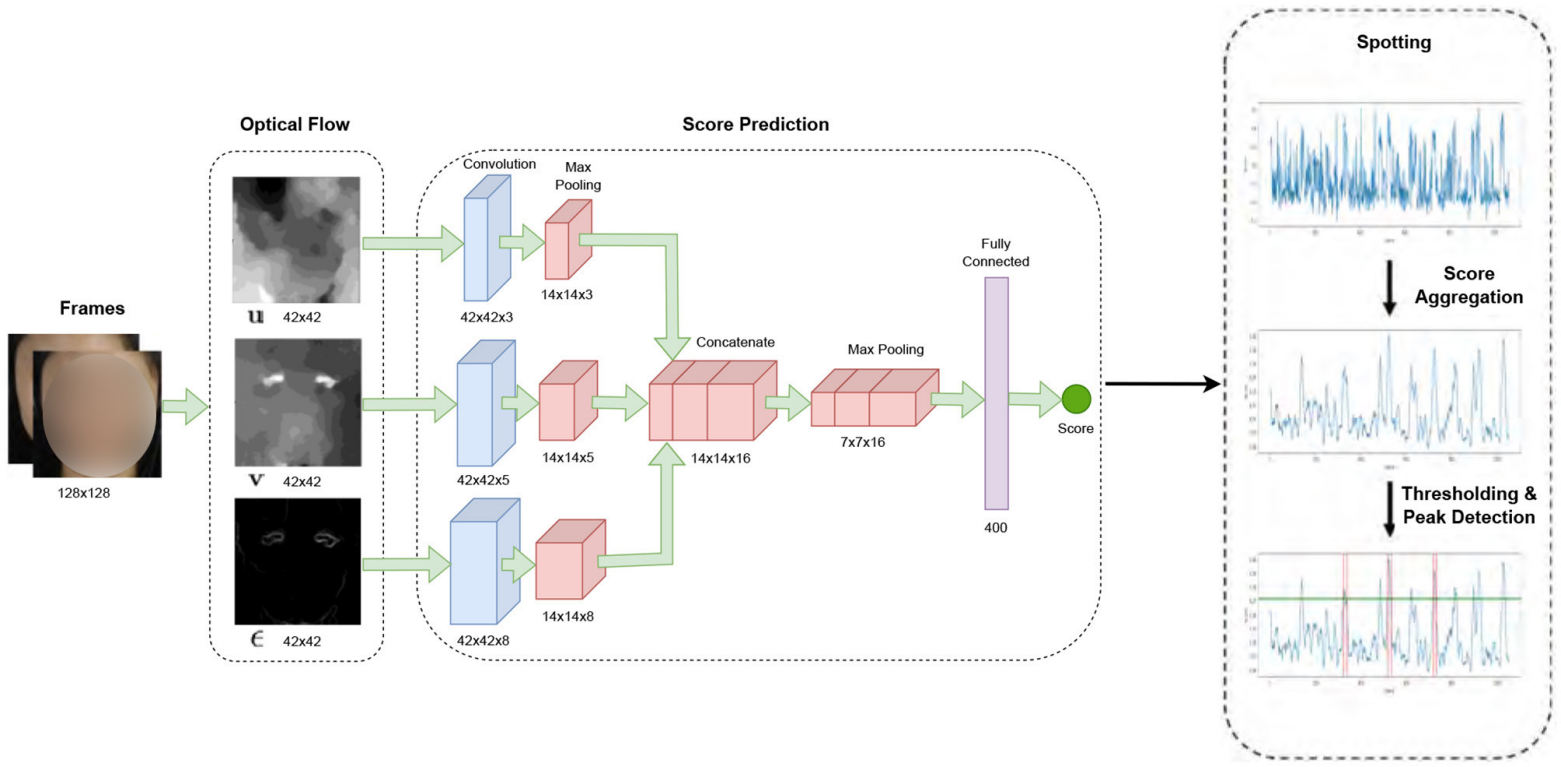
SOFTNet framework for micro-expression spotting (Liong et al., 2021).

In our study, micro-expression detection leveraged the Shallow Optical Flow Three-stream CNN (SOFTNet) technique (Liong et al., 2021), a pioneering method for distinguishing subtle facial movements in extended video footage. This method, grounded in analyzing micro-expressions—subtle, involuntary facial movements that reveal suppressed emotions, employs a novel shallow optical flow CNN architecture to discern these fleeting expressions. The SOFTNet model could predict the likelihood of a frame capturing a micro-expression interval, treating the detection challenge as a regression problem (Figure 3). This model assigned scores to each frame, reflecting its probability of belonging to a micro-expression. Frames with scores surpassing a certain threshold were identified as the apex frame of a micro-expression. Expanding on this approach, we used the model to spot micro-expressions across all videos, delineating micro-expression intervals. Each of these intervals spanned 30 frames, where the peak of the micro-expression was designated as the apex, and the initial frame marking the start of the expression was regarded as the onset.

#### 2.2.3 Extracting features from micro-expressions

**Figure 4:**
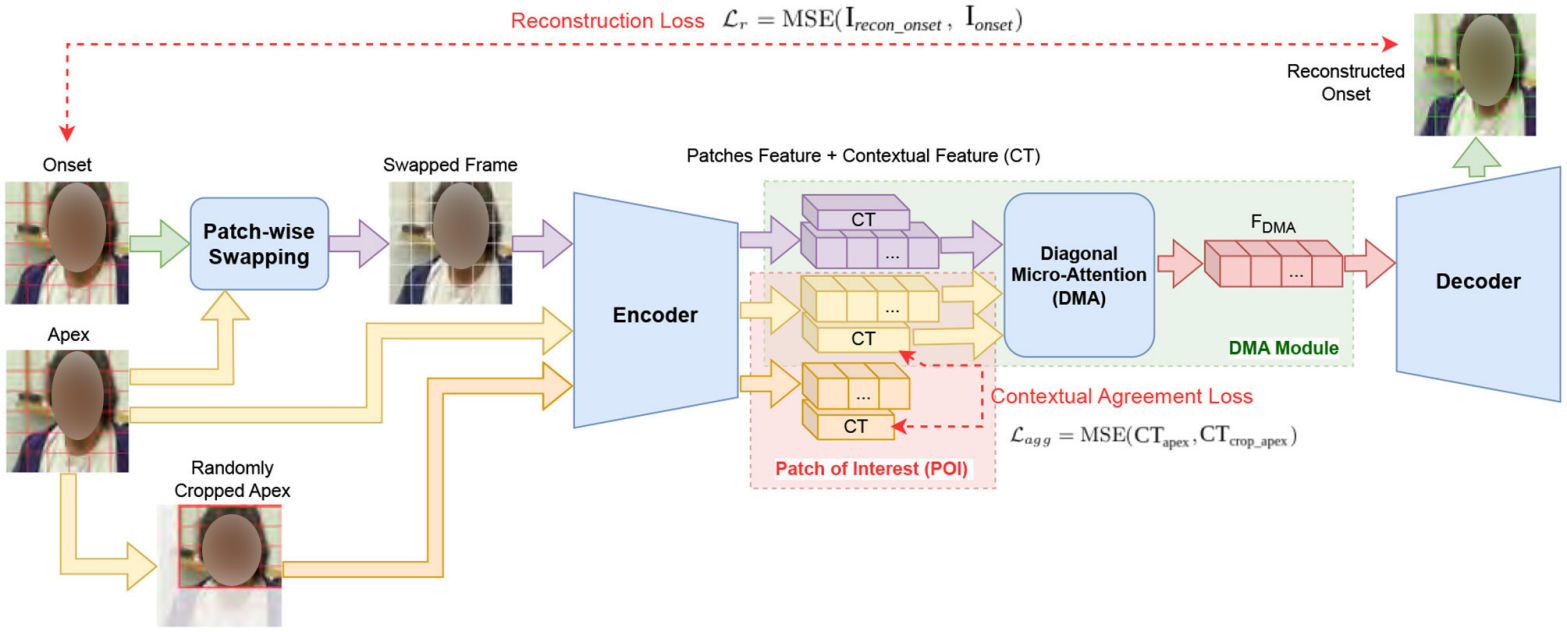
Micron-BERT framework for micro-expression feature extraction.

For each of the micro-expression intervals detected from SOFTNet, we leveraged the Micron-BERT technique (Nguyen et al., 2023) to extract micro-expression features. This approach was specifically designed to recognize and analyze subtle facial movements that are generally difficult for human observers to detect. Micron-BERT employs a sophisticated framework that incorporates several key modules: Blockwise Swapping, Patch of Interest (PoI), and Diagonal Micro Attention (DMA). The comprehensive framework of Micron-BERT is illustrated in Figure 4.

**Figure 5:**
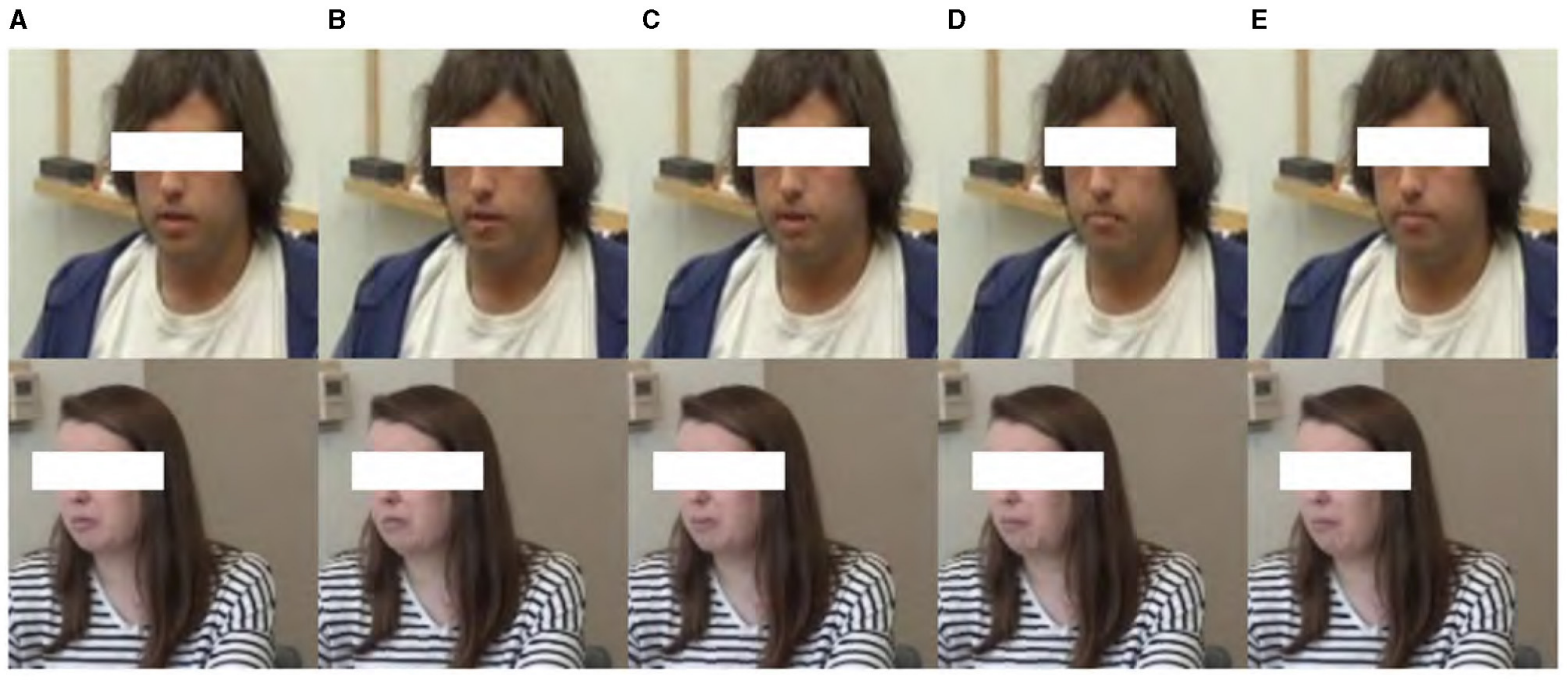
Samples of Patch-wise Swapping result with different swapping ratios: **(A)** 0, **(B)** 0.3, **(C)** 0.5, **(D)** 0.7, **(E)** 1.0.

**Patch-wise swapping**. This technique involved strategically swapping blocks of pixels, known as “patches,” between the apex and onset frames of a micro-expression. Each frame was divided into multiple patches, each with an 8 × 8 pixel block. These small, contiguous regions of the image were treated as individual units for analysis and manipulation, enabling precise control over the image data. By altering the content of these patches, we forced the model to concentrate on the differences these swaps introduce rather than on the original unaltered image context. This manipulation significantly challenged Micron-BERT to detect and adapt to the most subtle changes in facial expressions, which is crucial for accurately identifying micro-expressions. The swapping ratio, a critical parameter in this process, specified the proportion of the frame that underwent this swapping, influencing the model's sensitivity to dynamic changes in facial expressions. We experimented with swapping ratios—0, 0.3, 0.5, 0.7, and 1.0—to determine their impact on model performance. A swapping ratio of 0 means that the swapped frame was identical to the onset frame, while a ratio of 1.0 means that the swapped frame was identical to the apex frame. Adjusting this ratio allowed us to control the intensity of the disturbance and thereby control the model's sensitivity to dynamic facial changes. This method enhanced the robustness of the feature detection and helped better differentiate significant facial movements from normal variations. Figure 5 shows the swapping result with different ratios.

**Patch of Interest (PoI) module**. This module selectively focused on areas of the face that were most likely to exhibit micro-expressions. By isolating these regions, the PoI module effectively reduced background noise and distractions, enhancing the clarity and accuracy of the feature extraction process.

**Diagonal Micro Attention (DMA) module**. This mechanism was essential for detecting minute differences between the apex and swapped frames. It focused on these micro-variations by analyzing diagonal patterns in the attention maps, which helped pinpoint the precise location of micro-expressions within the facial regions.

By incorporating these elements, Micron-BERT effectively captured and analyzed the fleeting and subtle facial movements that characterized micro-expressions. The swapping between apex and onset frames enriched the model's training data, providing a robust basis for recognizing and classifying micro-expressions across different scenarios and individuals. This methodology elevated the precision of detecting micro-expressions and enriched the understanding of the emotional nuances expressed during these brief facial movements.

#### 2.2.4 Classification

##### 2.2.4.1 Defining classification problems

Our study focused on the following two primary binary classification tasks.

**ASD vs. controls**. This classification task involved distinguishing between individuals diagnosed with ASD and TD samples. This binary classification was crucial for evaluating how effectively our feature extraction method could identify behavioral markers that are diagnostically significant.

**RSI symptom item**. This classification task within the study was more nuanced, focusing on the presence or absence of specific symptoms within the ASD sample, as indicated by scoring on the respective ADOS-2 RSI items. In this task, we categorized the scores into two groups:

- Zero score (ZS): It represented RSI items scored as 0, indicating typical or near-typical behavior in social interactions. This suggested lesser or no signs of ASD-related impairments in the assessed interaction.
- Non-zero score (NZS): It represented RSI items scored as anything other than 0. This indicated atypical behavior, with scores reflecting varying degrees of social interaction challenges characteristic of ASD.

This classification task aimed to uncover underlying patterns in the RSI scores that could potentially indicate distinct ASD profiles or variations in characteristics.

##### 2.2.4.2 Comparison of MLP, SVM, and ResNet classifiers

We have compared three classifiers: multi-layer perception (MLP) (Werbos, 1974), support vector machine (SVM) (Cortes, 1995), and ResNet (He et al., 2016) with a linear kernel to classify features extracted by the DMA component of our Micron-BERT model. These three models have varying complexity for handling high-dimensional data. Depending on the resource constraints in practice, it is often unclear which model is the best for processing the nuanced features extracted by DMA, which captured subtle facial expressions indicative of emotional states and social responsiveness by linear kernels. In addition to the efficiency and effectiveness of the linear kernel in handling linearly separable data, another critical aspect that influenced our choice of using a linear kernel along with three classification models was its interpretability. This is particularly valued in clinical and diagnostic settings because it facilitates a clear understanding of the decision-making process, essential for clinical acceptance and further research implications.

The comparison between MLP, SVM, and ResNet with a linear kernel to classify features extracted by the DMA component of our Micron-BERT model can be found in **Table 5** of experimental results. To determine the final classification of a participant, we used a majority voting system: if more than half of a participant's intervals were classified as ‘positive”, then the overall diagnosis for the participant was ASD or NZS. Conversely, if most intervals were “negative”, the participant was classified as TD or ZS. This majority rule could be adjusted in its threshold to improve the sensitivity and specificity of the model, ensuring that our classification process aligned with the necessary clinical accuracy for diagnosing ASD. This voting mechanism consolidated findings from multiple intervals and enhanced the diagnostic outcome's robustness and reliability.

### 2.3 Statistical analysis and permutation tests

In evaluating the effectiveness of our model, we utilized the F1 score and accuracy as the primary metrics to ensure the reliability and accuracy of our findings. To ensure robustness and avoid overfitting, we implemented 10-fold cross-validation, where the dataset was divided into ten subsets, and the model was trained and tested across these subsets in a rotating fashion. This approach helped to provide a more generalized performance estimate. Additionally, to confirm that the model's predictive success was statistically significant and not due to random chance, we conducted permutation tests by shuffling the labels and comparing the model's performance under this scenario with the original unshuffled data. This statistical framework ensured that our model was reliable and effective in distinguishing between ASD vs. TD (or NZS vs. ZS) individuals, supporting its potential utility in clinical diagnostics.

### 2.4 Model explainability

#### 2.4.1 Significant patch visualization

In our analysis, we introduced the Significant Patch Visualization (SVP) technique to highlight areas within our data that significantly impacted our classification outcomes. This technique utilized the coefficients of a linear kernel SVM, which served as our classification model. By focusing on these coefficients, we could identify and visualize the top 1,000 features that played a crucial role in distinguishing between classes, specifically ASD vs. TD (or NZS vs. ZS) individuals.

**SPV**. We started by analyzing the weights assigned to each feature in the linear SVM. These weights indicated the importance of each feature in the classification process, with higher absolute values suggesting greater significance. From this analysis, we selected the top 1,000 features based on the absolute values of their coefficients. This subset of features was considered to have the most impact on the model's decision-making process. Each of these top features was traced back to their original location in the input data, allowing us to identify which patches of the data these features were derived from. We then created a heatmap representation of the input data, where each patch was colored based on the count of top features it contains. Patches with a higher concentration of significant features were highlighted more prominently.

**Mask test**. To further validate the effectiveness of our model, we conducted a mask test based on the SPV. In this test, specific regions of the input frames were deliberately obscured—the central facial area (Central Mask Test, C-MT) and the areas outside the central region (Non-Central Mask Test, NC-MT)—to remove targeted visual information. We could compare the outcomes by masking these areas separately and then classifying the modified frames with our model. This experiment helped us assess whether facial regions enhanced the accuracy of our classification.

#### 2.4.2 t-distributed Stochastic Neighbor Embedding

We also used t-SNE to explain our machine-learning model for ASD diagnosis. This technique reduces the high-dimensional data derived from the model into a two- or three-dimensional space, making it visually interpretable. By observing the clusters formed in the t-SNE plot, we gained valuable insights into the effectiveness of our feature extraction techniques. Well-defined clusters indicated that the model captures meaningful patterns distinguishing between various classes.

## 3 Results

### 3.1 Diagnostic classification: ASD vs. controls

The results of our study, as summarized in Table 3, demonstrate the effectiveness of machine learning models in distinguishing between individuals with ASD and TD controls across different swapping ratios. The table presents the accuracy (Accu.) and F1 Score (F1) for ASD vs. controls classification across five swapping ratios (0, 0.3, 0.5, 0.7, and 1.0) for various scenarios. The “Top 3” and “Top 5” indicate the scenario-level fusion based on the top three or five best-performing scenarios. In this experiment, “Top3” includes scenario 5 (work/school), scenario 11 (daily living), and scenario 13 (loneliness), and “Top5” additionally includes scenario 7 (emotions) and scenario 12 (relationships).

**Table 3:** Accuracy (%) and F1 Score of ASD vs. controls classification on Caltech and WVU ADOS video datasets.

**Swapping ratio**	**0**		**0.3**		**0.5**		**0.7**		**1.0**	
	**Accu**.	**F1**	**Accu**.	**F1**	**Accu**.	**F1**	**Accu**.	**F1**	**Accu**.	**F1**
Scenario 5	94.82	0.94	94.81	0.94	94.82	0.95	94.21	0.94	**94.82**	0.94
Scenario 6	89.82	0.89	89.82	0.89	88.57	0.87	89.82	0.89	89.82	0.89
Scenario 7	91.07	0.90	89.82	0.89	89.82	0.89	91.25	0.90	89.82	0.89
Scenario 11	94.58	0.94	94.58	0.94	93.33	0.93	94.58	0.94	93.33	0.93
Scenario 12	93.57	0.93	93.57	0.93	**96.07**	**0.96**	92.32	0.92	92.32	0.92
Scenario 13	94.46	0.94	94.46	0.94	94.46	0.94	94.46	0.94	94.46	**0.94**
Scenario 14	89.17	0.90	89.17	0.90	90.42	0.91	90.42	0.91	90.42	0.91
Top 3	**96.07**	**0.96**	**96.07**	**0.96**	94.82	0.95	**97.32**	**0.97**	**94.82**	**0.94**
Top 5	94.82	0.95	**96.07**	**0.96**	94.82	0.94	96.07	0.96	**94.82**	**0.94**
All	94.82	0.95	94.82	0.95	93.57	0.93	94.82	0.95	93.57	0.93

“Top3” and “Top5” indicate the scenario-level fusion based on the top three and five best-performing scenarios, respectively. “Top3” includes scenario 5 (work/school), 11 (daily living), and 13 (loneliness), and “Top5” additionally includes scenario 7 (emotions) and scenario 12 (relationships). The best results are highlighted by bold.

The data shows that the machine learning models maintained relatively high accuracy and F1 scores across most interview topics and swapping ratios. Topic 5 (work and school) consistently shows high performance, with the highest accuracy and F1 score peaking at 94.82%. Additionally, the performance peaks in the “Top 3” and “Top 5” scenario fusion underscore the highlighting of the model, especially at higher swapping ratios. The “Top 3” category achieved the highest recorded F1 score of 0.97 at a swapping ratio of 0.7, suggesting optimal performance when more facial features are swapped. In comparison, the overall performance across all scenarios (“All”) indicates consistent results with minor fluctuations in accuracy and F1 scores, maintaining above 93.57% and 0.93, respectively.

**Figure 6:**
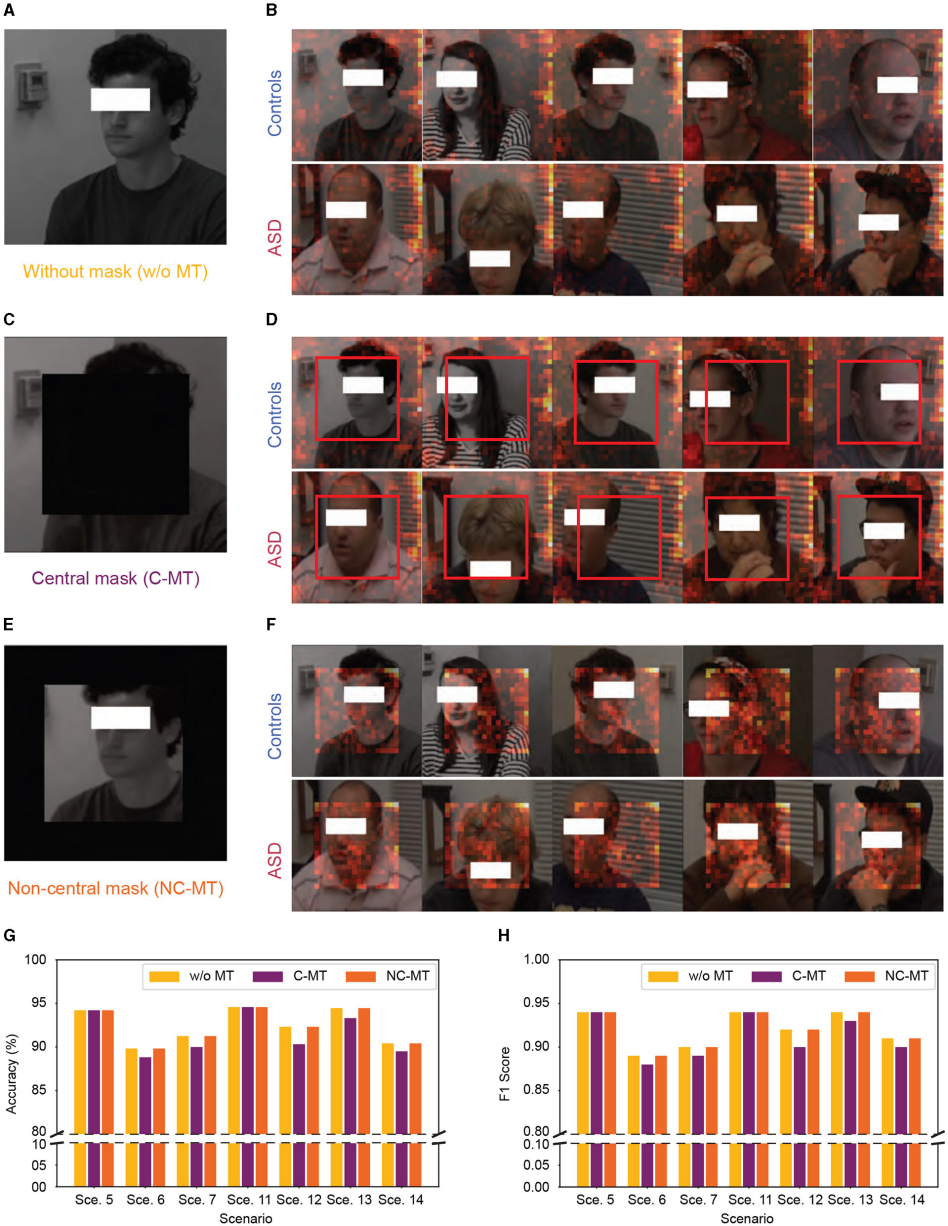
Significant patch visualization of the ASD vs. controls classification with and without masking. **(A, B)** Without mask test. **(C, D)** Central mask test (C-MT). **(E, F)** Non-central mask test (NC-MT). **(G, H)** Accuracy and F1 Score performance with 0.7 swapping ratio, “Sce.” indicates scenario. **(A, C, E)** Samples of input frames with and without mask. **(B, D, F)** Samples of significant patch visualization from ASD and controls. The red boxes in **(D)** highlight the absence of a significant patch in C-MT.

To clarify the underlying factors guiding our micro-expression model's classification decisions, we utilized SPV to highlight the specific areas the model prioritizes during classification. However, as illustrated in Figures 6A, B it became apparent that the model did not primarily rely on facial details for classification; instead, it concentrated on the peripheral regions of the images. To further verify whether the high performance of our model was genuinely associated with facial information, we conducted a mask test as shown in Figures 6C–F. The mask test results showed that masking the facial regions only slightly impacted the model's performance. As shown in Figures 6G, H, both the C-MT and the NC-MT resulted in the model maintaining high-performance levels comparable to those observed in the unmasked condition. This unexpected reliance on background features suggested that the model might not be learning the underlying patterns specific to ASD and control distinctions but exploiting dataset biases.

### 3.2 RSI symptom item classification

Table 4 shows the performance of classifying RSI items across various scenarios on the Caltech ADOS video datasets. Overall, the aggregated results for the “Top3” and “Top5” scenarios maintain higher average accuracy and F1 scores across all classifications, confirming the importance of these scenarios in the effective classification of ASD-related micro-expressions. Unlike the ASD vs. controls classification task, in this experiment, “Top3” includes the scenario 6 (social), 11 (daily living), and 13 (loneliness), and “Top5” additionally includes scenario 5 (work/school) and scenario 14 (plans/hopes). Across all RSI behaviors (B01–B13), scenario 11 (daily living) consistently achieves high accuracy and F1 scores, indicating its significance in identifying ASD-related micro-expressions. Additionally, RSI items B01 (unusual eye contact) and B03 (language production and linked nonverbal communication) achieve higher accuracy than other items.

**Table 4:** Accuracy (%) and F1 Score of the individual RSI item classification on Caltech ADOS video datasets.

**Scenario**	**B1**		**B2**		**B3**		**B4**		**B5**	
	**Accu**.	**F1**	**Accu**.	**F1**	**Accu**.	**F1**	**Accu**.	**F1**	**Accu**.	**F1**
5	83.33	0.81	38.33	0.36	83.33	0.81	61.94	0.55	42.78	0.40
6	85.56	0.79	43.06	0.41	85.56	0.79	67.22	0.64	50.28	0.50
7	83.93	0.80	**52.82**	**0.53**	83.93	0.80	**72.42**	**0.66**	50.67	0.46
11	**87.78**	**0.82**	44.48	0.44	**87.78**	**0.82**	65.95	0.61	51.79	**0.53**
12	85.83	**0.82**	40.83	0.40	85.83	**0.82**	64.17	0.56	**54.44**	**0.53**
13	87.70	**0.82**	51.98	0.51	87.70	**0.82**	61.15	0.57	51.98	0.49
14	87.22	0.81	34.33	0.27	87.22	0.81	68.17	**0.66**	41.63	0.42
Top3	83.06	0.78	45.00	0.45	83.06	0.78	68.89	0.65	52.50	**0.53**
Top5	85.28	0.81	45.28	0.43	85.28	0.81	61.94	0.58	50.28	0.52
All	85.28	0.81	45.28	0.45	85.28	0.81	59.44	0.56	50.00	0.51
**Scenario**	**B6**		**B7**		**B8**		**B9**		**B10**	
	**Accu**.	**F1**	**Accu**.	**F1**	**Accu**.	**F1**	**Accu**.	**F1**	**Accu**.	**F1**
5	**56.39**	**0.55**	56.94	0.56	36.11	0.33	63.33	0.62	55.00	0.55
6	47.22	0.46	63.89	0.62	45.28	0.42	63.61	0.60	58.61	**0.58**
7	34.60	0.31	**73.85**	**0.70**	34.05	0.33	63.85	0.58	52.18	0.52
11	45.71	0.46	68.53	0.65	41.63	0.39	61.51	0.57	**59.96**	0.56
12	54.44	0.54	61.39	0.59	49.44	0.46	64.44	0.61	52.22	0.51
13	42.94	0.43	69.05	0.67	45.52	0.42	**74.76**	**0.68**	47.34	0.43
14	45.79	0.45	67.34	0.65	**58.10**	**0.56**	65.04	0.59	50.32	0.48
Top3	42.78	0.41	66.39	0.65	49.44	0.47	64.44	0.61	51.94	0.51
Top5	52.50	0.53	66.67	0.64	47.50	0.47	68.89	0.66	47.50	0.47
All	54.72	**0.55**	59.67	0.58	40.56	0.40	61.67	0.60	52.20	0.51
**Scenario**		**B11**			**B12**			**B13**		
		**Accu**.		**F1**	**Accu**.		**F1**	**Accu**.		**F1**
5		61.67		0.58	38.33		0.38	33.89		0.35
6		59.44		0.56	**56.94**		**0.58**	52.22		0.52
7		52.18		0.50	52.90		0.52	42.54		0.41
11		63.45		0.58	46.43		0.47	50.71		0.49
12		59.44		0.57	43.06		0.41	37.50		0.38
13		65.60		0.58	36.35		0.35	49.29		0.48
14		59.29		0.53	43.29		0.41	43.69		0.41
Top3		**66.94**		**0.63**	47.50		0.48	**56.67**		**0.57**
Top5		**66.94**		**0.63**	45.28		0.45	54.72		0.55
All		57.22		0.53	42.78		0.43	47.78		0.48

“Top3” includes the scenario 6 (social), 11 (daily living), and 13 (loneliness), and “Top5” additionally includes scenario 5 (work/school) and scenario 14 (plans/hopes). The best results are highlighted by bold.

**Figure 7:**
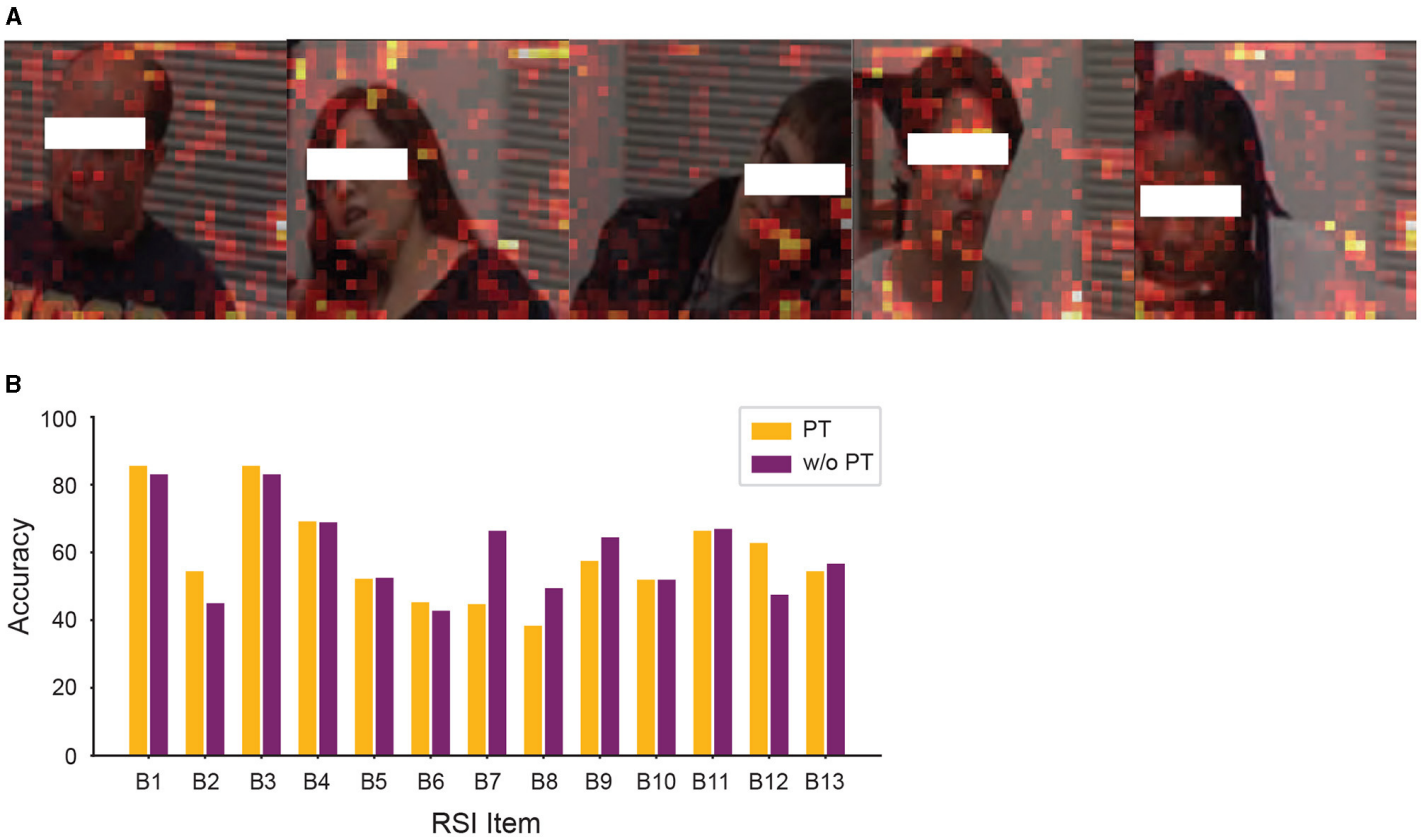
**(A)** Significant patch visualization of the individual RSI item classification. **(B)** Performance comparison between the permutation test (PT) and without the permutation test, showing the accuracy from “Top3” includes the scenario 6 (social), 11 (daily living), and 13 (loneliness).

However, constrained by the issue of data imbalance, especially in items B01 and B03, we further utilized SPV to highlight the specific areas that the model prioritizes during classification, as shown in Figure 7A. As the SPV experimental results indicate, we found that the basis for classifying RSI items was relatively random and not concentrated on facial areas. Additionally, we employed permutation tests to examine the model's validity, as shown in Figure 7B. According to the permutation tests, the model's performance in micro-expression classification for most items was comparable to the permutation test results, with item B07 (insight into typical social situations and relationships) being the only exception demonstrating a relatively higher performance improvement.

The superior performance in item B07 can be attributed to several factors. First, B07 involves a deep understanding of social situations and relationships, which requires processing subtle social cues and emotions. Micro-expressions, as brief facial expressions that reveal genuine emotions, provide critical insights into spontaneous reactions and understanding of these social dynamics. This makes micro-expressions a valuable biomarker for evaluating insight into social relationships. Second, understanding social situations often involves recognizing and interpreting complex emotional states. Individuals with ASD may display distinct micro-expressions that reflect their differences in emotional processing. The model's ability to capture these subtle variations enhances the accuracy of ASD diagnosis, particularly for an item like B07, which relies heavily on emotional and social cognition. Lastly, providing examples of social insight spontaneously means that individuals will likely show genuine emotional responses. Micro-expressions are less likely to be masked or controlled than other forms of expression, reflecting more accurately the participant's true social understanding. This authenticity in emotional response is crucial for distinguishing between typical and atypical social cognition, thereby improving the model's performance for item B07.

We also compared the SVM with other classifiers for individual RSI item classification on the Caltech ADOS video datasets using the “Top3” scenario fusion, as shown in Table 5. SVM achieved the highest average accuracy across items (MLP: 58.50%, ResNet50: 59.10%, SVM: 59.89%), outperforming both MLP (Werbos, 1974) and ResNet50 (He et al., 2016) in some RSI items, particularly in B01 (unusual eye contact), B03 (language production and linked nonverbal communication), B04 (shared enjoyment in interaction), B07 (insight into typical social situations and relationships), and B13 (overall quality of rapport), where it recorded the highest accuracy and F1 scores. While ResNet50 demonstrated competitive accuracy in some items like B06 (comments on others' emotions/empathy) and B09 (quality of social overtures), MLP generally underperformed compared to SVM and ResNet50, especially in items like B02 (facial expressions directed to examiner), B06, and B08 (responsibility). Overall, SVM's superior performance highlights its suitability for this classification task.

**Table 5:** Accuracy (%) and F1 Score comparison of the SVM with other classifiers for individual RSI item classification on Caltech ADOS video datasets using “Top3” scenario fusion.

**Classifier**	**B01**		**B02**		**B03**		**B04**		**B05**	
	**Accu**.	**F1**	**Accu**.	**F1**	**Accu**.	**F1**	**Accu**.	**F1**	**Accu**.	**F1**
MLP (Werbos, 1974)	78.33	0.75	40.56	0.39	78.06	0.75	59.44	0.55	**61.67**	**0.61**
ResNet50 (He et al., 2016)	78.61	0.75	**47.78**	0.44	78.89	0.75	61.67	0.54	57.50	0.56
SVM (Cortes, 1995)	**83.06**	**0.78**	45.00	**0.45**	**83.06**	**0.78**	**68.89**	**0.65**	52.50	0.53
**Classifier**	**B06**		**B07**		**B08**		**B09**		**B10**	
	**Accu**.	**F1**	**Accu**.	**F1**	**Accu**.	**F1**	**Accu**.	**F1**	**Accu**.	**F1**
MLP (Werbos, 1974)	37.78	0.38	64.17	**0.65**	**50.28**	**0.49**	61.94	0.61	**56.94**	**0.57**
ResNet50 (He et al., 2016)	**57.22**	**0.53**	59.44	0.58	47.78	0.40	**71.11**	**0.64**	42.22	0.40
SVM (Cortes, 1995)	42.78	0.41	**66.39**	**0.65**	49.44	0.47	64.44	0.61	51.94	0.51
**Classifier**		**B11**			**B12**			**B13**		
		**Accu**.		**F1**	**Accu**.		**F1**	**Accu**.		**F1**
MLP (Werbos, 1974)		**69.17**		**0.65**	**50.00**		**0.50**	52.22		0.52
RestNet50(He et al., 2016)		64.44		0.58	47.22		0.44	54.44		0.55
SVM (Cortes, 1995)		66.94		0.63	47.50		0.48	**56.67**		**0.57**

The best results are highlighted by bold.

### 3.3 Remove input data background

**Figure 8:**
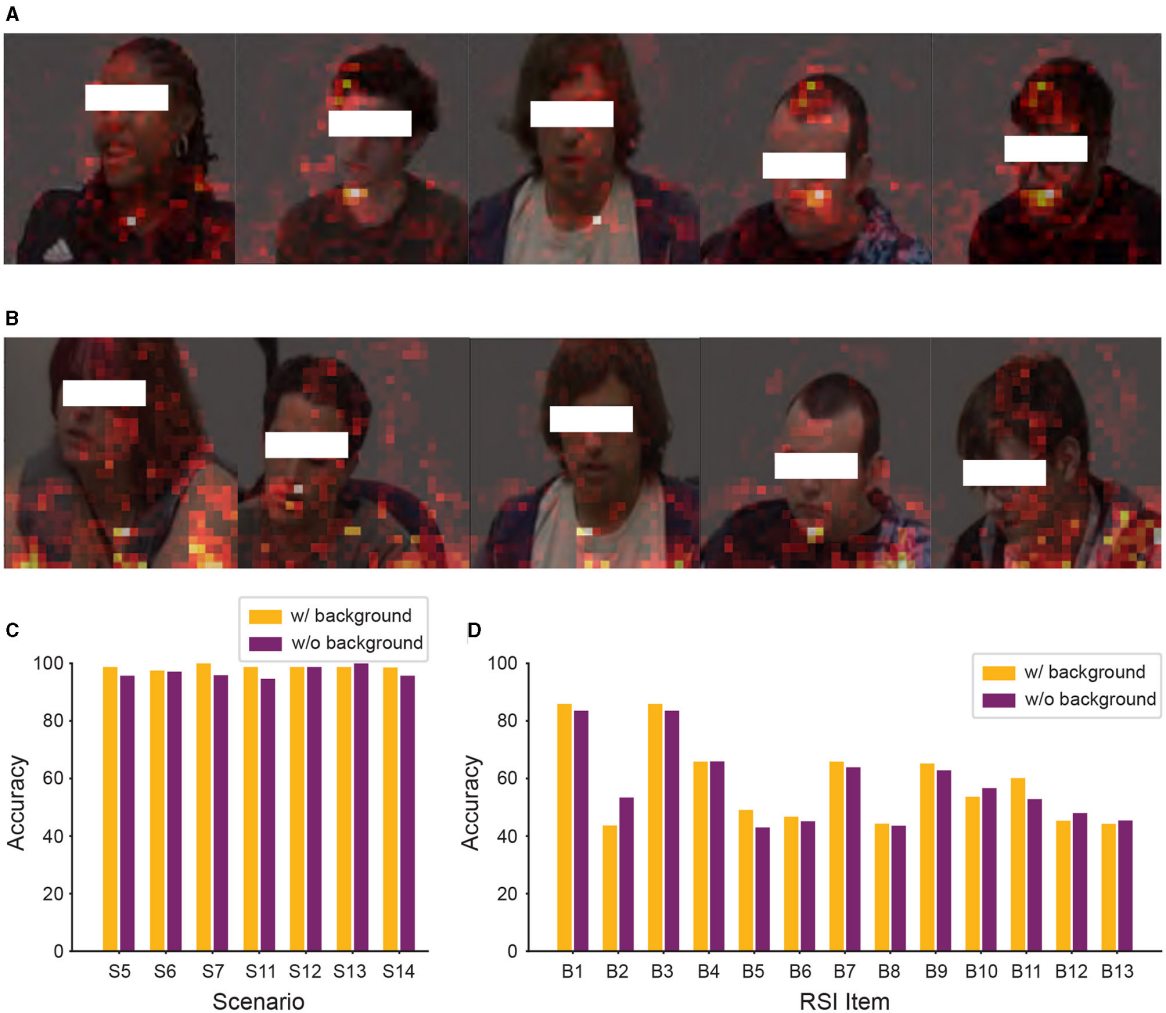
Significant patch visualization of the RSI item classification with or without background. **(A, C)** ASD vs. controls classification. **(B, D)** Individual RSI item classification. **(C)** Performance comparison across different RSI items, showing the average accuracy calculated across the seven scenarios for each item. **(D)** Performance comparison on the 13 individual RSI items.

From Figure 7A, we observed that background information still affected our model's classification. To further improve the performance of our model, we removed the background information from the input data of Caltech and WVU ADOS video datasets. After this adjustment, the experimental results are shown in Figure 8. As shown SPV in Figures 8A, B, the model focused more on human regions in both classification tasks, as indicated by the clear outlines of people. This suggests that our model's classification basis is concentrated on human areas, due to the removal of background information, allowing it to better target relevant human features. However, as shown in Figures 8C, D, despite this adjustment, the comparison of model performance in both classification tasks revealed that removing background information did not consistently improve accuracy. In some experimental settings, it even lowered performance, particularly in the ASD vs. controls classification task, where most scenarios showed a decline in accuracy.

### 3.4 t-SNE

**Figure 9:**
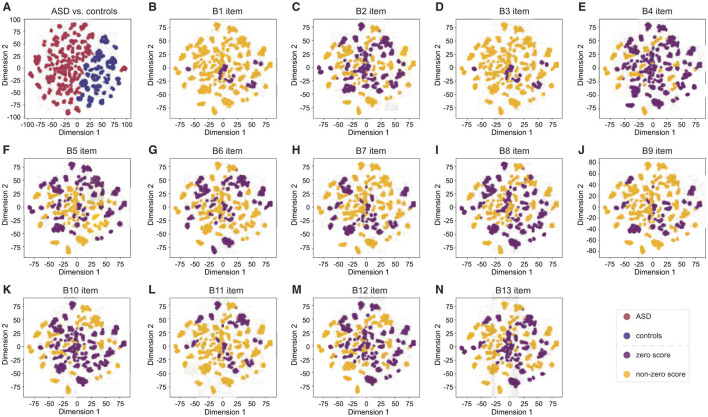
Visualization in the t-SNE space for extracted micro-expression features. **(A)** ASD vs. controls classification. **(B–N)** Individual RSI item classification (B1–B13 item).

Figure 9 provides t-SNE visualizations for the extracted micro-expression features, comparing ASD vs. controls classification (Figure 9A) and individual RSI item classifications (Figures 9B–N).

In the ASD vs. controls classification (Figure 9A), we observe a clear separation between ASD (red points) and controls (blue points). This indicates that the model can distinguish between these two groups but for reasons likely unrelated to the true differences in micro-expressions. The t-SNE visualizations and accuracy comparisons with the mask test reveal that the model's high performance in ASD vs. controls classification is primarily due to learning the biases between the two databases rather than capturing the underlying micro-expression patterns that differentiate ASD individuals from controls.

Several issues are evident for the individual RSI item classifications (Figures 9B–N). There is a significant overlap between zero score points (purple) and non-zero score points (orange) for all RSI items. This indicates that the features extracted for these items are not distinct enough to provide a clear separation between zero and non-zero item scores. Such overlap suggests that the model struggles to effectively differentiate between the micro-expressions associated with the behaviors reflected in these score. Specifically, in Figure 8B (B1 unusual eye contact item), clustering non-zero score points around zero score points leads to a lack of clear feature separation, likely leading to classification challenges. Similar overlap patterns are observed in other items, where the indistinct clustering further explains the lower performance in these classifications.

## 4 Discussion

Can micro-expressions be used as a biomarker for ASD? Experimental findings in this report seem to support the negative answer for the following reasons.

- Reliability of micro-expression detection: Despite the use of Micron-BERT (Nguyen et al., 2023) (the current SOTA), its reliability in detecting micro-expressions depends on various uncertainty factors such as video quality and inductive bias. Without fine-tuning the Micron-BERT on our prioritized dataset, its performance has not been optimized.
- Expression vs. micro-expression: It is important to contrast the experimental results in this report with our previous work for facial expressions (Zhang et al., 2022; Zhang, 2023). Due to the transient nature and short duration of micro-expressions, it is plausible that facial expressions contain more discriminative information needed to distinguish ASD from the control group.
- Clinical relevance of micro-expressions to ASD: Micro-expressions have not been clinically shown to be directly related to autism. It was hypothesized that the non-stationary stochastic patterns of minute fluctuations (micro-movements) (Torres et al., 2013) might facilitate the diagnosis of ASD. However, the subtle patterns in micro-expressions of infants with ASD (e.g., the smiling-related) are often beyond layperson's naked eyes (Alvari et al., 2021).

However, as sensing and computing technologies co-evolve, micro-expression datasets with higher quality and resolution might change our view in the future. Here, we suggest a few lines of research for future study of ML for autism video.

- Video data quality: High-resolution and high-frame-rate videos are essential for capturing micro-expressions, which are brief and subtle (typically spanning only 1/25 to 1/5 of a second). If the video quality is poor, critical details may be missed, reducing the ability of machine learning models to detect and analyze these expressions accurately.
- Variability in expressiveness: Individuals with ASD exhibit a wide range of expressiveness. Some may display fewer facial expressions naturally, or their micro-expressions may differ from typical patterns, making standard detection algorithms less effective. Future research should focus on developing detection algorithms that are more sensitive and tailored to these unique patterns of expressiveness in individuals with ASD.
- Complexity of micro-expressions: Micro-expressions are complex and can be extremely subtle, often requiring advanced imaging techniques and sophisticated analytical tools for detection, which may not yet be refined enough for clinical ASD diagnostics. Future research should focus on refining these tools and techniques to improve their sensitivity and reliability, making them more suitable for clinical ASD diagnostics.
- Multimodal collaboration: Micro-expression alone might not be qualified as a biomarker. Still, it does not necessarily imply that it cannot be used in conjunction with other known biomarkers to facilitate the diagnosis of ASD. For example, children with ASD are known for the lack of response to name-calling—we hypothesize that a multimodal system that focuses on micro-expression following the event of name-calling might convey more discriminative information about the children's behavior in joint attention settings.

## Data Availability

The data analyzed in this study is subject to the following licenses/restrictions: only accessible to users that have been approved by IRB. Requests to access these datasets should be directed to shuowang@wustl.edu.
